# Status of information, education, and communication as perceived by clients receiving antenatal care at Chiradzulu District Hospital in Malawi

**DOI:** 10.1186/s12905-023-02209-2

**Published:** 2023-02-09

**Authors:** Gaily Graysham Lungu, Elizabeth Chodzaza, Martha Kamanga, Wanangwa Chikazinga, Diana Jere

**Affiliations:** 1 Department of Midwifery, School of Maternal, Neonatal and Reproductive Health, Kamuzu University of Health Sciences, P/Bag 360, Chichiri, Blantyre 3., Blantyre, Malawi; 2 School of Nursing. Department of Nursing Education, Kamuzu University of Health Sciences, P/Bag 1, Capital City, Lilongwe Malawi; 3 School of Nursing, Department of Mental Health, Kamuzu University of Health Sciences, P/Bag 360, Chichiri Blantyre 3, Malawi

**Keywords:** Information, Education, Communication, Antenatal care, Pregnancy

## Abstract

**Background:**

Information, education, and communication is a strategy to spread awareness through communication channels to a target audience to achieve a desired positive result. Women are supposed to receive information, education, and communication at each contact with the health worker during antenatal care. In Malawi, information, education, and communication for antenatal care is inadequate despite high antenatal care coverage. Most women do not receive it as stipulated. This could be one of the reasons that maternal and neonatal mortality is high. The provision of information, education, and communication is supposed to help in reducing maternal mortality because it is intended to develop positive attitudes towards health behaviours to support pregnant women accessing health services when required. This study, therefore, assessed the status of information, education, and communication as perceived by clients receiving antenatal care at Chiradzulu District Hospital in Malawi.

**Methods:**

A descriptive study design with a sample of 384 pregnant women attending antenatal care was used. The sample size for the study was calculated using Lemeshow, Hosmer, Klar and Rwanga's formula. Systematic random sampling method was used to select the study participants. Data were analysed using a statistical package for social sciences software version 20.0.

**Results:**

Findings revealed that information, education, and communication provided during antenatal care were inadequate. Most information was offered. However, no topic was rated adequate by 80% of the respondents according to the Likert Scale that was used. The majority of the respondents (71.4%, n = 274) (95% CI 66.5. 75.8) preferred to receive information, education, and communication from midwives who are in the category of skilled attendants. Results further showed that more than half of the respondents participated passively and spent little time receiving information, education, and communication.

**Conclusion:**

The findings signify that information, education, and communication provided to women receiving antenatal care at Chiradzulu District Hospital had some gaps. It was inadequate and some topics were not taught. The target audience participated passively. It is recommended that midwives should provide the information, education, and communication and must have adequate contact time with the women. This is so because they are believed to be trusted sources of information.

**Supplementary Information:**

The online version contains supplementary material available at 10.1186/s12905-023-02209-2.

## Introduction/Background

As part of antenatal care (ANC), pregnant women receive information, education, and communication (IEC) [1, 2]. IEC is a strategy to spread awareness through communication channels to a targeted audience to achieve a desired positive result. Regular IEC programs, whether provided individually or in groups during antenatal care, result in positive changes in people's health practices resulting in a healthy mother and baby [3].

Pregnant women who receive IEC as part of ANC are more able to recognize pregnancy hazard symptoms like vaginal bleeding (antepartum hemorrhage), convulsions, severe headaches with blurred vision, fever, difficulties in breathing, severe abdominal pains and backache and report to health facility once they suspect or experience any of the symptoms. [4–7]. IEC provided during ANC also enhances women's ability to plan for many aspects of care to satisfy their pregnancy's needs. Furthermore, IEC raises women's knowledge of potential pregnancy difficulties and influences their decision to seek competent delivery assistance [5, 8]. According to certain research, IEC offered to pregnant women during antenatal care is one of the most important variables in reducing maternal morbidity and mortality [3, 8]. Every day, almost 800 women die from pregnancy or childbirth-related problems around the world. Almost all of these maternal deaths (99 percent) occur in developing nations, with emerging countries accounting for more than half of all maternal deaths [8].

Malawi's maternal mortality rate is now projected to be 439 per 100,000 live births [9], which is considered too high compared to Zambia, a neighboring country in the sub-Saharan region which was at 278 per 1000 live births in 2018 [10]. Despite a downward trend from 749 in the year 2000 to 439 in 2017 [11]**,** it fell short of the United Nations Millennium Development Goals' objective of 155/100,000 live births in 2015, indicating systemic inequality [12].

Following the establishment of the Sustainable Development Goals (SDG) for 2030, governments have united around a new aim to reduce maternal mortality to less than 70 deaths per 100,000 live births [10]. As a strategic measure, Malawi must develop and scale up strong clinical practices to meet the maternal health targets, which include IEC during antenatal care [3]. In Malawi, IEC is one of the major components of ANC. Malawi adopted the WHO 8 contact model of antenatal care in the year 2019 which emphasizes more contacts between pregnant women and health professionals that lead to positive pregnancy outcomes [13]. This model can reduce perinatal deaths by up to 8 per 1000 births [10, 14]. Women are supposed to receive IEC during each of the contacts of which the first one is supposed to be done in the first trimester when the woman is about 12 weeks pregnant.

Chiradzulu District Hospital in southern Malawi is one of the hospitals which has the highest number of antenatal attendances within the region [9]. Its antenatal department provides IEC and currently utilises the 8-contact model of antenatal care. However, the status of the IEC that is provided is not known as no studies were done This study, therefore, aimed at assessing the status of IEC as perceived by clients during antenatal care clinic at Chiradzulu District Hospital in Malawi. This study addressed the global and national health priority of maternal mortality which is quite high in Malawi.

## Methodology

### Conceptual framework

Studies have indicated that the status of IEC given to pregnant women is what influences the outcomes [3, 15, 16]. It has to be of good quality. Much as the concept of quality is difficult to define, and is an abstract term, Donabedian defined high-quality care as the type of care that is offered to a patient in totality. This includes welfare while considering the potential gains and losses in all areas [17, 18]

**Fig. 1:**
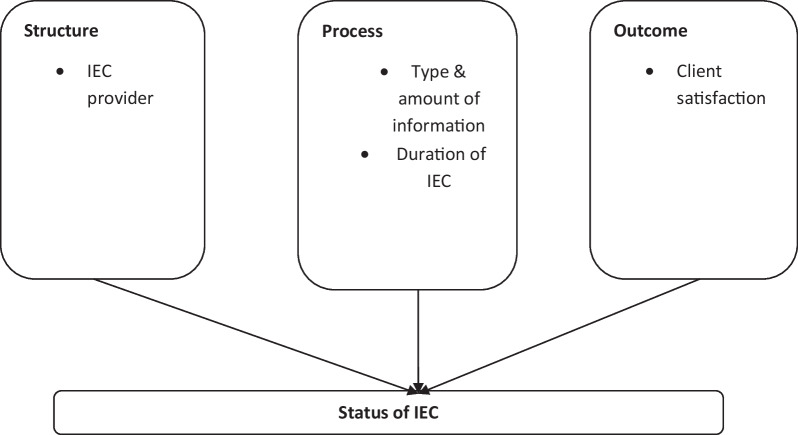
Modified Donabedian SPO Model. Adapted from Donabedian, (1980)

Donabedian suggests three components of quality care: structure, process, and outcome. He wrote that quality care should not be interpreted in the absence of a relationship among the three components such that structure influences process and process influence outcome in a more complex manner than a direct relationship [18]. The status of IEC was assessed following the three components. The structure described the context in which care is delivered, including the staff who provide the services. Process denotes the transactions between patients and providers throughout the delivery of healthcare. Finally, outcomes referred to the effects of healthcare on the health status of patients and populations. The status of IEC as perceived by clients receiving antenatal care was assessed following Donabedian’s components of quality where the providers were assessed under the structure. The process comprised of type and amount of information given to pregnant women as well as the duration of IEC. Client satisfaction was assessed on the outcome (Fig. 1).

### Study setting

The study was conducted at Chiradzulu District Hospital which is located in the southern region of Malawi. It is situated north-east of Blantyre, at 26.7 km from Blantyre City. The city has Queen Elizabeth Central Hospital where Chiradzulu hospital refers to complicated cases for further diagnosis and management. The district had approximately 15,916 expected pregnancies in 2015/2016 and 1192 pregnant women within the hospital’s catchment area [9]. The facility also receives clients from some locations of Blantyre district. About fifty women report for ANC every day at the facility. The facility provides essential health care services and acts as a secondary-level facility to 11 government health centres, 1 mission and 1 Islamic health centre, and a mission hospital. All these facilities provide IEC.

### Study design

A descriptive, cross-sectional study design [19] was used to assess the status of IEC offered during ANC at Chiradzulu District Hospital for one month from 31st May to 28th June 2016. Data were collected based on independent and dependent variables. Independent variables included participants’ age, marital status, educational level, occupation, residence, and gravidity. Type and amount of information offered, duration of IEC, and client satisfaction fell under dependent variables.

### Population and sample

The study targeted pregnant women aged 18–49 years, who have had two or more pregnancies and had reported for ANC at the hospital. The study excluded pregnant women of less than 18 years of age and primigravida’s because most women including young primigravida’s (24%) start ANC in the second or third trimester [20] instead of 12 weeks as recommended by WHO antenatal care model. Such women may not be exposed to IEC [21]. This is the same reason why women with first-time pregnancies were excluded as well. Those who were unable to comprehend the consenting process were excluded too (see Additional file 1: appendix 1). The study used a systematic random sampling technique to select study participants [22, 23]. The total sample size of 384 was calculated using a formula proposed by Lemeshow, Hosmer, Klar & Lwanga (1990) which stipulates that n = Z^2^ (p) (1−p) /e^2^ and interpreted as follows: n = sample size, Z = value of a normally distributed variate, which for 95% confidence interval takes the value of 1.96 and p = estimated proportion of antenatal women to the number of women of reproductive age from within the catchment area. Because the specific proportion was not known, 0.5 was used for the calculation. e = desired precision or a standard error was set at 0.05. Therefore, the calculation according to Lemeshow, et al. (1990) was done as follows:

n = Z^2^ (p) (1–p)/e.^2^

n = [1.96^2^ (0.5) (1–0.5)]/0.05^2^

n = [3.8416 × 0.5 × 0.5]/0.0025.

n = 384.16

n = 384

Therefore, 384 participants were recruited.

The participants were selected by dividing the population of pregnant women within the catchment area with the target sample size to come up with the Kth member which is every third woman attending ANC and meeting the criteria.

### Data collection and analysis

A structured questionnaire developed by the researcher after reviewing various literature on IEC and antenatal care was used for data collection (see Additional file 2: appendix 2). Some parts of the questionnaire were adapted from Integrated Maternal and Neonatal Health (IMNH) manual for Malawi. The manual is a competency-based document used by practicing midwives and other skilled birth attendants. The manual equips them with knowledge, skills, and appropriate attitudes in the management of life-threatening conditions during pregnancy, delivery, and the postpartum period, as well as basic essential newborn care [24]. The manual has necessary IEC topics to be offered to pregnant women during ANC. Before the actual data collection, the instrument was pretested on 10% of the total sample at the antenatal department of Thyolo District Hospital to assess the feasibility of the study and to test the accuracy and reliability of the questionnaire. Data were obtained through face-to-face interviews with two research assistants who underwent an orientation of the data collection tool and procedures. Face-to-face interviews were done to ensure the quality of obtained data and increase the response rate. Researchers collected data from all the respondents in a private room at the antenatal clinic by administering the questionnaire verbally. The administration of the questionnaire by the researchers ensured that all respondents understood the information that was being asked thereby assisting those who had problems with comprehension. About 30 min was used for collecting data from each respondent. The structured questionnaire assisted the researcher to be systematic and ensure that similar questions are asked to all participants [25]. The questionnaire was used to collect appropriate data, to make questions engaging and varied, and to minimize bias in formulating and asking questions. There were very few incomplete questionnaires which were replaced by feasible and completely new ones. For data management and participant privacy, each questionnaire was given a number for easy identification. Data were kept in a lockable cabinet which was only accessed by the research team.

Data were analysed using SPSS version 20.0. However, the type of IEC in the questionnaire was compared with the IMNH standards for Malawi based on Likert scale (adequate, not adequate, not provided, and no idea). Participants were asked whether they received education on each topic. A topic on which 80% and above of the respondents rated it adequate was regarded as of good status. Good status or quality may be defined as the degree to which health services for individuals and populations increase the likelihood of desired health outcomes [26]. In this case, the perception of trust among recipients of care was positively regarded [27].

## Results

### Demographic characteristics of respondents

The study recruited 384 women who reported to Chiradzulu District Hospital for ANC and a response rate of 100% was achieved since the sample size was reached. The majority of the participants were aged between 25 and 34 with a mean age of 27.55 years and a standard deviation of 5.5. At least ¾ of the respondents were married and more than half attended primary education with 36.2% (n = 139) who reached secondary education. Most of the participants, 67.2% (n = 258), lived in rural areas and 16.7% (n = 64) lived in semi-urban areas.

The results of the study further showed that 43.2% (n = 166) of the respondents were housewives, who were not doing any business and 33.3% (n = 128) of them were doing small-scale businesses. The number of pregnancies (gravidity) for a majority of the respondents 93% (n = 360) was 2–4 (see Table 1).

**Table 1:** Distribution of respondents by demographic characteristics

Characteristics	Frequency (n = 384)	Percentage (%)
*Age*		
18–24	135	35.2
25–34	206	53.6
35–44	42	10.9
45–49	1	0.3
*Marital status*		
Not married	56	14.6
Married	320	83.3
Divorced	6	1.6
Separated	2	0.5
*Education level*		
None	5	1.3
Primary	218	56.8
Secondary	139	36.2
Tertiary	22	5.7
*Occupation*		
Not working	166	43.23
Civil servant	30	7.81
Business	128	33.33
Other	60	15.63
*Residence*		
Semi-urban	64	16.7
Rural Chiradzulu	258	67.2
Other	62	16.1
*Gravidity*		
2	122	31.8
3	132	34.4
4	75	19.5
5–8	55	14.3
*Number of visits*		
First	66	17.2
Second	139	36.2
Third	108	28.1
Fourth	59	15.4
Fifth	12	3.1

### Structure

#### People who provide IEC

**Fig. 2:**
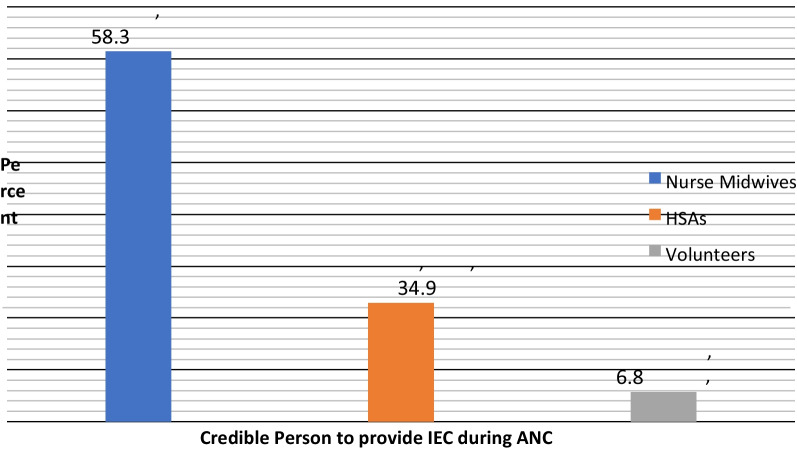
Percentage of IEC providers during ANC

About 58.3% (n = 224) of the respondents mentioned Nurse Midwives as people who provide IEC during ANC while 34.9% (n = 134) mentioned Health Surveillance Assistants. Volunteers were the least mentioned cadre that provided IEC. The majority of the participants 71.4% (n = 274) (95% CI 66.5. 75.8) preferred to receive IEC from Nurse Midwives. See Fig. 2.

### Process

#### Topics taught during ANC

The IMNH Manual [20] stipulates the topics that should be offered during IEC. Findings from this research revealed that several topics were offered at ANC on daily basis. According to the respondents, symptoms of labour, danger signs in pregnancy, plans for delivery, diet and nutrition, and effects of STIs were the topics most likely to have been discussed (see Table 2).

**Table 2:** Topics taught during antenatal care

Adequate n (%)	Not adequate n (%)	Not provided n (%)	No idea (%)
Process of pregnancy and its 152 (39.6) complication	124 (32.3)	102 (26.5)	6 (1.6)
Diet and nutrition 250 (65.1)	113 (29.4)	21 (5.5)	
Rest and exercise in pregnancy 160 (41.6)	117 (30.5)	102 (26.6)	5 (1.4)
Personal hygiene 254 (66.1)	126 (32.8)	1 (0.3)	3 (0.8)
Danger signs in pregnancy 268 (69.8)	102 (26.5)	6 (1.6)	8 (2.1)
Use of drugs in pregnancy 211 (55)	149 (38.8)	17 (4.4)	7 (1.8)
Effects of STIs/HIV 262 (68.2)	119 (31)	3 (0.8)	
Exclusive breastfeeding 247 (64.3)	116 (30.2)	10 (2.6)	11 (2.9)
Symptoms/signs of labour 271 (70.6)	103 (26.8)	4 (1.0)	6 (1.6)
Importance of colostrum, early 233 (60.7) initiation	132 (34.3)	13 (3.4)	6 (1.6)
Plans for delivery (birth 266 (69.3) preparedness)	110 (28.6)	8 (2.1)	
Plans for postpartum care 215 (56)	132 (34.4)	31 (8)	6 (1.6)
Family planning 174 (45.3)	122 (31.8)	81 (21.1)	7 (1.8)
Harmful habits (e.g., smoking, 142 (37) drug abuse, alcoholism)	97 (25.3)	121 (31.5)	24 (6.2)

#### Duration of IEC

Respondents were asked about the duration for IEC to clients on their initial visit to the antenatal clinic. About half of the women reported that IEC to initial clients took more than 20 min. and, 42.2% (n = 162) said that clients who reported for subsequent antenatal care did not receive IEC.

### Outcome

#### Client satisfaction with IEC

About 71.6% (n = 275) of the respondents reported that they were satisfied with the IEC offered during ANC, while 28.4% (n = 109) of the respondents reported that they were not satisfied. About 63.3% (n = 243) of the respondents were satisfied with the venue where IEC is offered and the amount of information offered.

## Discussion

The WHO and the Ministry of Health for Malawi recommends that women should receive information, education, and communication at each visit during antenatal care [24, 28]. However, some information is not offered to women during antenatal care. In this study, we assessed the status of IEC as perceived by clients receiving antenatal care at Chiradzulu District Hospital in Malawi. We found that IEC provided at Chiradzulu antenatal department had some gaps. Most information offered during antenatal care was not adequate and women preferred to receive IEC from midwives who are in the category of skilled health professionals. Midwives as well as other health workers have the responsibility to inform women about the care they should expect.

The survey discovered that almost all women who took part in the study went to primary school, with some who went up to tertiary education. The findings are consistent with what the NSO found in 2017. The NSO found that 54.5 percent of women in Malawi have completed some type of primary education [9]. This makes individuals more receptive to health care, especially the information provided during antenatal care. Education is critical because it enhances people's comprehension of concerns and care, as well as their receptivity to healthcare communications [29].

Furthermore, education improves health-seeking behavior because educated women are more likely to seek care and have access to the best healthcare options for themselves and their children [5]. This variable also has an impact on women's social standing, which has been shown to influence antenatal care uptake decisions [30].

Health workers can plan a teaching strategy that is appropriate for the pregnant women present based on their educational level. They should always check antenatal women's educational levels to ensure that they grasp the IEC being presented and support those who may have difficulties in understanding the content [31]. The majority of the women in the research were housewives who did not work. Women rely on their spouses for financial support. As a result, even if they obtain IEC during ANC, women may still rely on their husbands to make health decisions for them [5].

### Structure

From this study, we found that Nurse Midwives and other support health personnel like Health Surveillance Assistants (HSA) are the most likely people who provide IEC to Chiradzulu antenatal clinic. However, the study revealed that the IEC is given a little time than recommended by focused antenatal care (FANC) guidelines [3]. This is in relation to the findings of a study that nurses who are among the highest cadres of skilled attendants have a shorter interaction time with pregnant women when providing IEC [32]. In addition, most of the respondents preferred to receive IEC from Nurse Midwives. It is common knowledge that individuals prefer hearing about health issues from their Nurse Midwives or Clinicians with a feeling that a health worker is a reliable source of information [31].

### Process

Some of the recommended topics for IEC according to the IMNH manual for Malawi are offered during antenatal care at Chiradzulu district hospital. The following topics were being offered during ANC; diet and nutrition, personal hygiene, danger signs in pregnancy, effects of STIs and HIV/AIDS in pregnancy, exclusive breastfeeding, and symptoms of labor and plans of delivery. However, among the list of topics that need to be offered during antenatal care as suggested by the IMNH manual for Malawi [24] no topic was rated as adequate by 80% of the respondents according to the Likert scale that was used. This concurs with the previous studies which report that important subjects such as diet and nutrition, family planning, and danger signs in pregnancy are not adequately taught [15, 16].

On the other hand, the study noted that topics like harmful social habits in pregnancy, importance of rest and exercises during pregnancy, family planning, and process of pregnancy and its complications were not taught at any of the visits. Most study participants were living in rural areas. NSO, (2017) and Anya (2008) write that women living in rural areas are the least likely to receive information on pregnancy complications than those living in urban areas [9, 15], the view which has been supported by the findings of this study.

IEC during ANC is important as it provides pregnant women with vital information on issues such as decision-making, skills for labor, post-natal care, and parenting skills. Pregnant women are also supposed to be informed and educated on alcohol and tobacco use, safe sex, rest, sleeping under insecticide-treated nets (ITNs), birth and emergency plan, post-natal care, infant feeding, and family planning [33].

The FANC model recommends 30–40 min for the first visit and 20 min for subsequent visits to carry out all activities including IEC [3, 34]. IEC alone should take about 15 min according to the simulation of FANC [3]. We found that women who reported for initial and subsequent antenatal care did not receive the required education. The findings of the study correspond with other studies that found that the average time currently spent for providing ANC service to a first-visit client was 5 min instead of 45 min as recommended by FANC [1, 3]. Such type of communication can be a challenge and it suggests poor interaction between the provider and the client. Providing IEC separately on initial and subsequent clients is good as it avoids repetition and reduces the delay of clients reporting for subsequent care since they only receive additional information to the one offered during previous visits [2]. Clients attending antenatal care on the initial visit are supposed to receive antenatal education as well as counseling services which takes a longer time.

In the same vein, Nwaeze et al. [1], in a study on perception and satisfaction with the quality of antenatal care services report that the moral relationship between midwife and patient or client is a substantial element of good practice. This is not only because it identifies problems quickly and clearly but it also defines expectations and helps establish trust between the provider and the patient. Spending less time with antenatal women may compromise the IEC because inadequate information is provided.

The results of this study further showed women participated passively by listening during the IEC with few who participated actively by answering and asking questions. Giving women chance to participate during IEC helps to clarify misunderstandings about the information provided. The lack of active participation by the women during IEC may signify a lack of understanding of the given information. Consistent with the literature, this research found that some women grew forceful to address their needs as a result of poor communication, while others became hesitant to actively interact with providers [4].

### Outcome

The study showed that although women were satisfied with the information provided during antenatal care, the level of satisfaction that women had did not correspond with the services that were offered because of the shortfalls in the type and of information given, and the limited time that was allocated. Previous researchers revealed that the level of satisfaction was not always in accordance with a willingness to access services [1, 16]. Women may generally express satisfaction with the antenatal services despite the inconsistency between the IEC offered and facility expectation [1]. This may mean that women do not know what to expect from health care providers or their expectations on the care to be provided is low. Therefore, women need to be well informed about the services they should expect from health care providers and be able to ask for it if not provided [35].

Health workers also have the responsibility to inform women about the care that they should expect [16]. Despite this responsibility, health workers are overwhelmed and it is challenging because not every woman’s needs are addressed and counselling could be compromised because of large numbers of women reporting for ANC. Considering that the WHO eight contact model for antenatal care [28] could also increase workload for midwives, there is need for Ministry of Health to consider increasing more health workers in antenatal departments or use other approaches like group antenatal care which targets women of same gestation age group for effective provision of services.

## Conclusion

The findings of this study signify that IEC provided at Chiradzulu antenatal department has some gaps. Women reported that most information offered during antenatal care was not adequate and they preferred to receive IEC from midwives who are skilled health professionals. This study recommends that health workers should allow adequate time and ensure proper interaction with the clients to get more information from them which in turn will assist in information, education, and counseling offered to the clients. Health workers also have the responsibility to inform women about the care they should expect.

## Limitations of the study

The study was done at one hospital and results may not be generalized to the whole country with different contextual factors. On the other hand, the status of IEC was perceived by recipients of care. There is a need to do a similar study to find out from the perspectives of midwives and other health workers who offer antenatal services. Furthermore, the study needs to be replicated in other regions since the findings cannot be generalized as no similar studies have been done so far. Excluding the participants, less than 18 years old is a challenge for this study since their input could have contributed to the results.

## Supplementary Information

**Additional file 1. Appendix 1:** Information sheet for pregnant adolescent.

**Additional file 2. Appendix 2:** Questionnaire for pregnant women.

## Data Availability

All data supporting this study is provided as Additional files 1 and 2 accompanying this paper.

## Abbreviations

IEC: Information Education and Communication
ANC: Antenatal care
MMR: Maternal Mortality Rate
SPSS: Statistical Package for Social Sciences
IMNH: Integrated Maternal and Neonatal Health
FANC: Focused Antenatal Care
NSO: National Statistical Office
ITN: Insecticide Treated Net
WHO: World Health Organization
