# Supplementary material for: Status of information, education, and communication as perceived by clients receiving antenatal care at Chiradzulu District Hospital in Malawi
Source: BMC Womens Health. 2023 Feb 9;23:53. doi: 10.1186/s12905-023-02209-2 (PMC9909957; doi:10.1186/s12905-023-02209-2)
Supplement: Supplementary file 1 — Additional file 1. Appendix 1: Information sheet for pregnant adolescent. [file 12905_2023_2209_MOESM1_ESM.pdf]

Information Sheet for Pregnant Women

Dear Madam / Sir,

My name is Gaily Lungu, currently a student at Kamuzu College of Nursing, a constituent college of the University of Malawi. I am pursuing a Master's Degree in Midwifery. I am required to conduct a research study in partial fulfillment of my Master of Science degree in midwifery program. My research study is on *"assessing quality of information, education and communication during antenatal care at Chiradzulu District Hospital*". Its aim is to explore the views of pregnant women as recipients of care on the information they receive during ANC. This letter therefore serves as a request for you to participate in this research.

Your participation will involve providing information regarding the respected areas on the questionnaire. You may wish to know that your participation in the study will not have any reasonably foreseeable risks or discomfort to you**.** However, in case you experience any physical or emotional harm please forward your concerns to COMREC Secretariat, P/Bag 360, Chichiri, Blantyre 3, Telephone number 01 871911 extension 334.

There are no direct benefits in participating in the study, however, your views are important because they will assist service providers to understand the status of information, education and communication that needs to be provided during antenatal care. This in turn will help to reduce maternal and neonatal morbidity and mortality. However, the findings of the study will be shared to you upon your wish.

Whatever information you provide will be kept strictly confidential and will not be shown to other people except the researcher and other people who are directly involved in the research. You will not be asked a name instead codes will be used. However, little background information will be obtained from you in order to form part of the database.

Participation in the study is voluntary and there is no penalty for refusing to take part. You may choose to participate or not, or to withdraw from the study at any time. Your refusal to participate or withdrawal from the study will not have any negative effects on you as well as the antenatal services that are offered to you at this hospital. Should you agree to participate in this study, I will ask you to sign a consent form in order to indicate that you have voluntarily accepted to be interviewed.

The study and its procedures have been approved by College of Medicine Research and Ethics Committee (COMREC), and Chiradzulu District Hospital authorities. If you have questions or you need clarifications about this study, you can contact me on +265 888 63 40 12 / + 265 996 48 11 33 or you may raise your concerns to COMREC Secretariat, P/Bag 360, Chichiri, Blantyre 3, Telephone number 01 871911 extension 334.

Thank you for taking your time to read this information letter.

Consent Form for Pregnant Women

I have read or have had another person read to me the information given by the researcher above and have understood the content of the information, its aim, procedures and the expected duration of my participation. I have been given an opportunity to ask questions about the study where necessary. I understand that the information that I will share with the researcher will be kept confidential and will only be accessed by the researcher and those people who are directly concerned with the study.

I understand that I will not have any direct benefits for participating in the study but that the findings of the study will assist service providers to understand the status of information, education and communication that needs to be included during antenatal care. This in turn will help to reduce maternal and neonatal morbidity and mortality. I know that neither do I have to suffer any injury or harm during the research process nor that the information I will give to the researcher will be used against me in future. I also know where to complain if my rights are violated during the study. I am aware that participation is voluntary and that I am free to withdraw from the study at any time without being penalized.

I voluntarily agree to participate in the study.

Participants signature…………………………………………………...Date…………………

Participant’s thumbprint (if illiterate)……………………………………...Date …………………

Signature of witness (if participant illiterate)……………………………...Date………………….

Researcher’s signature………………………………………………….Date………………….
