# Supplementary material for: Status of information, education, and communication as perceived by clients receiving antenatal care at Chiradzulu District Hospital in Malawi
Source: BMC Womens Health. 2023 Feb 9;23:53. doi: 10.1186/s12905-023-02209-2 (PMC9909957; doi:10.1186/s12905-023-02209-2)
Supplement: Supplementary file 2 — Additional file 2. Appendix 2: Questionnaire for pregnant women. [file 12905_2023_2209_MOESM2_ESM.pdf]

1. Appendices

Appendix 1: Questionnaire for Pregnant Women

Date of interview……………………… Participant's identification number……………

Starting time………………………… Finishing time………………………

Part A: Demographic Data 1.

1. How old are you?

………………………………

2. What is your marital status?

   a) Married [ ]

   b) Single [ ]

   c) Divorced [ ]

   d) Widow [ ]

   e) Separated [ ]

   f) Other (specify) ………………………

3. How far have you gone with your education?

   a) I have never been to school [ ]

   b) Primary level [ ]

   c) Secondary level [ ]

   d) Tertiary level [ ]

4. Where do you stay?

   a) Chiradzulu district central (semi urban) [ ]

   b) Rural Chiradzulu [ ]

   c) Other (specify) ………………………

5. What is your occupation?

   a) House wife [ ]

   b) Civil servant [ ]

   c) Business lady [ ]

   d) Others specify……………………………..

6(i) What is your gravidity? ………………………… 6(ii) Parity? ………………….

7. What is your gestation age? …………………………………..

8. Which Antenatal visit is this?

a. First visit [ ]

b. Second visit [ ]

c. Third visit [ ]

d. Fourth visit [ ]

e. Other (specify) ………………………

Part 2: IEC Offered during ANC

Available Resources used for Provision of IEC

1. At the hospital, who provide IEC?

a. Nurse Midwife

b. Health Surveillance Assistant

c. Volunteer

d. Other (specify) ……………………………………………………..

2. Which of these sources is most credible to you?

a. Nurse Midwife, [ ]

b. Health Surveillance Assistant [ ]

c. Volunteer

e. Other (specify)..

3. (i) Are you actively involved during IEC sessions?

a. Yes [ ]

b. No [ ]

4(ii) If yes, how?

a. By sharing any information I know regarding the topic being discussed[ ]

b. By asking question [ ]

c. By answering questions [ ]

d. Other (specify)…………………………………..

Topics covered during IEC and amount of information given on each topics given to pregnant women during ANC

5. How much is the following information provided at ANC?

	**Adequate**	**Not adequate**	**Not provided at all**	**No idea**
a. Process of pregnancy and its complication				
b. Diet and nutrition				
c. Rest and exercise in pregnancy				
d. Personal hygiene				
e. Danger signs in pregnancy				
f. Use of drugs in pregnancy				
g. Effects of STIs/HIV				
h. Exclusive breastfeeding				
i. Symptoms/signs of labour				
j. Importance of colostrums, early initiation				
k. Plans for delivery (birth preparedness)				
l. Plans for postpartum care				

m. Family planning				
n. Harmful habits (e.g., smoking, drug abuse, alcoholism)				
o. Schedule of return visits				
p. Other (specify)				

6. Do women on initial and subsequent visit have their IEC together?

   a) Yes [ ]

   b) No [ ]

7. How long is the IEC session take during first visit?

   a. Less than 5 minutes [ ]

   b. 5-10 minute [ ]

   c. 11 – 20 minutes [ ]

   d. Over 20 minutes [ ]

   e. Other (specify) ……………………

8. How long is the IEC session take during subsequent visit?

a. Less than 5 minutes [ ]

b. 5-10 minute [ ]

c. 11 – 20 minutes [ ]

d. Over 20 minutes [ ]

e. Other (specify) [ ]

Women's Satisfaction towards IEC Offered during ANC

9. As a recipient of care, are you satisfied with IEC offered during ANC?

a. Yes [ ] b. No [ ]

10. Which of the areas below are you satisfied regarding IEC offered during ANC?

a) Type of service provider offering IEC [ ]

b) Type of information offered [ ]

c) Amount of information provided [ ]

d) Duration of provision of IEC [ ]

h. Other (specify).........................

11. If you have a chance to make a choice on where to seek ANC, would you love to come back to Chiradzulu District Hospital?

    a) Yes [ ] b. No [ ]

12. What suggestion do you have for improving IEC at Chiradzulu District Hospital?

    a) Should be given on one on one

    b) Should be given in cubicles

    c) More time should be set aside for IEC

    d) Topics should be based on individual concerns

    e) Others (specify) …………………………………………………
